# Pure Water

**Published:** 1876-10

**Authors:** 


### *PURE WATER.*

Timothy L. Lynch, of No. 2 Harrison Street, New York, has invented and patented a Domestic Still, by the aid of which pure water is supplied for domestic purposes. As he charges us with having given him the idea that such a thing was needed, we are glad to notice the fact of its existence in these pages.

Pure water is a prime necessity of the highest form of animal life, and any means which will place it within the reach of all classes of people will be hailed with enthusiasm by medical men. It is well known by all who reflect, that absolutely pure water can only be obtained by the process of distillation—that is, by evaporation and condensation. Filters are at best very poor substitutes for vaporizers, as they merely strain out the particles held in suspension, leaving the water precisely as it would be if allowed to stand and deposit such foreign substances as are not dissolved by it. When the manufacturer of a filter tells the public that absolutely pure water is produced by the use of his strainer of charcoal, or sponge, or sand, or pebbles, or anything else, he states a fallacy, since no strainer is capable of purifying water from soluble albumenous substances, or other organic matter held in solution. In fact, the vaunted filter may become a source of danger and disease, since it is likely under ordinary usage to become so clogged and saturated with dirt, as to still further contaminate the water which passes through it. We had a filter in use the past summer until we became utterly disgusted with it, and sent it into retirement. It came to us highly recommended, but it proved worthless as a purifier after the first day. It was valuable for an hour or two, but the croton water soon deposited sufficient *débris* to clog the straining materials so that no water could pass through. It proved that our drinking water was horrible, but it was impotent to lessen the evil. We have felt very anxious for the introduction of a cheap and useful still for families, and trust Mr. Lynch's device will supply this much needed want. With such an appliance in general use, we feel certain that many disorders of the digestive organs will disappear. The best and purest water in use still lacks much of absolute purity, and much that is drawn from old wells is dangerously vitiated by proximity to vaults, and the admixture of sewage and other vile refuse. The cure for many devastating ills will surely come from an ample supply of distilled water for drinking, and in the preparation of food.

It is curious to notice how important events come about. On page 410 of the JOURNAL OF HEALTH for 1875—the December number—in an article headed "What Shall we Drink," we stated that the time would come when every house would have its apparatus for the manufacture of pure water. We declared that an excellent field existed for the inventor who should provide a boiler with condenser-attachment, for stoves and ranges, capable of keeping up the process of distillation whenever the fire is in operation, and thus supply pure water in abundance for the uses of the household. Our article met the eye of Mr. Lynch, and he went to work to supply the need. After many experiments and much labor, he produced a successful Still, and brought it to us for examination. He told us that we had inspired him to the work; that our editorial article had convinced him that a simple system by which pure water could be produced, was not only a necessity, but also, that such a device was possible. So he studied up, tested and patented it, and now has it on exhibition at the Fair of the American Institute, where it may be seen in the good work of distilling—not poisonous alcohol, but the purest of pure water.

Mr. Lynch has appointed the HEALTH FOOD Co., 137 Eighth Street, New York, agents for the sale of his Still, and it is to be hoped that a large sale will speedily be secured.

